# Changes in the oral environment after placement of lingual and labial orthodontic appliances

**DOI:** 10.1186/2196-1042-14-28

**Published:** 2013-09-11

**Authors:** Luca Lombardo, Yildiz Öztürk Ortan, Özge Gorgun, Chiara Panza, Giuseppe Scuzzo, Giuseppe Siciliani

**Affiliations:** Postgraduate school of Orthodontics, University of Ferrara, Via Montebello, 31, Ferrara, 44121 Italy; Department of Orthodontics, Istanbul University, Istanbul, Turkey; Incirli Caddesi, Hur sokak 1/5 Bakirkoy, Istanbul, Turkey

**Keywords:** Oral hygiene habits, Orthodontic treatment, Salivary test, Lingual appliance, Labial appliance

## Abstract

**Background:**

This study compared the oral hygiene and caries risk of patients treated with labial and lingual orthodontic appliances throughout a prospective evaluation of the status of the oral environment before and after bracket placement.

**Methods:**

A total of 20 orthodontic patients aged 19 to 23 years were included in the study and were divided into two groups: 10 patients wore Roth labial appliance (American Orthodontics, Sheboygan, WI, USA) and 10 patients wore STb lingual appliance (Ormco Corporation, Glendora, CA, USA). Plaque index (PI), gingival bleeding index (GBI), salivary flow rate, saliva buffer capacity, salivary pH, and *Streptococcus mutans* and *Lactobacillus* counts in saliva were determined at three time points: before orthodontic appliance placement (*T*0), 4 weeks after bonding (*T*1), and 8 weeks after bonding (*T*2). After appliance placement, all patients were periodically educated to the oral hygiene procedures. Wilcoxon rank and Mann-Whitney *U* tests were used to determine intragroup and intergroup differences as regards qualitative data. To compare quantitative data between the groups, chi-square and Fisher's exact tests were undertaken, while intragroup differences were tested with McNemar test. The level of statistical significance was set at *p* < 0.05.

**Results:**

Statistical analysis of the data obtained revealed a statistically significant difference between the data of *T*0 and *T*1 and the data of *T*0 and *T*2 of the PI scores and between *T*0 and *T*2 of the GBI scores in the group treated with the lingual appliance. The GBI value increased significantly between *T*0 and *T*1 but decreased significantly between *T*1 and *T*2 (*p* < 0.01) in the group treated with labial appliance. *S*. *mutans* counts increased significantly between *T*0 and *T*2 in the saliva samples of patients treated with lingual appliance. No statistically significant differences were found between *S*. *mutans* and *Lactobacillus* counts at the three terms of saliva collection in patients treated with labial appliance. No statistically significant differences were found between the two groups at the three time points as regards the salivary flow rate and saliva buffer capacity.

**Conclusions:**

Lingual and labial orthodontic appliances showed a different potential in modifying the investigated clinical parameters: patients wearing STb lingual orthodontic appliance had more plaque retention 4 and 8 weeks after bonding, while there were more gingival inflammation and more *S*. *mutans* counts 8 weeks after bonding. No differences were found between the two groups as regards the *Lactobacillus* counts, the salivary flow rate, and saliva buffer capacity.

## Background

If a patient needs an orthodontic treatment, it should be of real benefit to him/her, by improving occlusion, dental and periodontal health, the longevity of dentition, speech, appearance, and self-esteem. Sometimes, we also have to consider patients' requirements for an esthetic or invisible treatment that is nowadays offered by aligner technology or lingual techniques. It was in 1979 when Fujita published the first article on lingual appliance, a real progress for the orthodontic practice [1]. Adults and teenagers liked better this innovative and attractive appliance above all for its esthetics [2]. It was then assumed that this appliance provoked less white spot lesions or caries development than the conventional labial appliance with obvious esthetic implication for the patient, thanks to less plaque retention due to the mechanical cleaning of the tongue on the lingual surfaces of the teeth [3]. Moreover, the fixed lingual appliance was also advisable for patients presenting some labial demineralization before starting with orthodontics. We must consider that white spots on the labial side of the teeth can occur rapidly, within the first month of therapy.

Thus, it is important to know what changes may occur in the oral environment during therapy before deciding if and how to treat a malocclusion. However, like many other interventions, orthodontic treatment has inherent risks and complications [4, 5], for example, oral hygiene may be difficult to maintain during treatment, which may lead to plaque accumulation, gingival inflammation, and enamel demineralization. Caries is a common complication in orthodontics, affecting 2% to 96% of all orthodontic patients [5]. Increased caries risk during the treatment is due to several factors:

- Lesions are difficult to locate,
- Lowering of resting pH,
- Increased volume of dental plaque,
- Rapid shift in bacterial flora [6]–[8].

Therefore, patient selection and the need for caries risk assessment at the beginning of the treatment play a vital role in minimizing risks of the treatment, so the clinician should be vigilant in assessing every aspect of the patient and his/her malocclusion [9]–[11].

Considerable time and effort has been spent on developing tests to identify individuals at risk for dental caries and periodontal damage. Different tests exist based predominantly on the quantitative estimation of *Streptococcus mutans* and *Lactobacillus* and on the determination of quantity and quality of saliva [12]–[14]. Positive correlations between caries activity test score and the counts of *S*. *mutans* and *Lactobacillus* have been reported [15].

In the literature, there are many studies investigating oral hygiene and caries risk during orthodontic treatment with labial fixed appliances [4]–[11, 16]–[25], but only few are evaluating the situation with lingual techniques [1, 26]–[34], and none has evaluated the situation with labial versus lingual appliances in the same people sample.

The aim of this study was to compare microbiological and clinical findings in patients treated with labial and lingual orthodontic appliances throughout a prospective evaluation of the status of the oral environment before and after bracket placement, by analyzing salivary and bacterial risk markers.

## Methods

The present study selected 20 patients aged 19 to 23 years who responded to the following criteria:

1. No systemic disease,
2. No antibiotic or antibacterial mouthwash usage within the last 1 month,
3. Periodontal health,
4. No caries or demineralization,
5. All permanent dentition.

The 20 non-extraction class I patients were randomly divided into two experimental groups: 10 patients (8 females, 2 males; mean age 19.3 ± 3.6) wore Roth labial metal brackets (American Orthodontics, Sheboygan, WI, USA) and the other 10 (7 females, 3 males; mean age 22.3 ± 3.2) wore STb lingual brackets (Ormco Corporation, Glendora, CA, USA), both with a 0.018-in × 0.025-in slot system. After enamel etching, upper and lower first molar tubes and brackets were bonded in both groups with Maximum Cure (Reliance Orthodontic Products, Itasca, IL, USA). The ligature system was based on preformed 0.010-in stainless steel ligatures. After the approval of the local ethical committee and the written informed consent of the patients, clinical and microbiological evaluations were collected at three time points: before the placement of the orthodontic appliance (*T*0), 4 weeks after bonding (*T*1), and 8 weeks after bonding (*T*2). After the placement of the appliances, all of the patients were periodically educated to brush their teeth three times a day, according to the modified Bass technique [35], using a manual toothbrush (Oral B, Procter & Gamble, Cincinnati, OH, USA) and fluoride toothpaste only. The study design involved two researchers: researcher A calibrated *ad hoc* to give oral hygiene instructions and to get clinical records and researcher B was instructed to adjust the appliances. According to the WHO guidelines for permanent dentition [36], the study registered the following:

- *Decayed*, *missing*, *and filled teeth* (*DMFT*) *index*. It collects the number of decayed missing, and filled teeth in comparison with the total examined teeth [36].
- *Plaque index* (*PI*). It is recorded by circulating a periodontal probe between the bracket base and free gingival margin at six sites around each tooth; if plaque deposits are found, the site is positive [37].
- *Gingival bleeding index* (*GBI*). It records the possible bleeding produced by probing the gingival sulcus of the gingival margin with a periodontal probe; if it bleeds within 10 s after probing, the site is positive [37].

Plaque and gingival bleeding Index scores are expressed as percentages of the total number of tooth surfaces examined. Stimulated saliva was collected by chewing paraffin gum for 5 min and expectorating into a sterile cup. Stimulated saliva samples were used to determine the following:

- *Salivary flow rate*. It is low if <0.7 ml/min and normal or high if ≥0.8 ml/min.
- *Salivary buffer capacity and pH*. It was obtained by mixing 1 ml of saliva with 3 ml 0.005 N HCl in a tube and measuring the solution pH after 10 min using color indicator strips (Merck KgaE, Darmstadt, Germany). It is low if pH <4.4, borderline/limited if pH ≤4.8, and normal if pH = 5.8 to 7.2.
- *Streptococcus mutans count*. Saliva (1 ml) was placed in 3 ml of transport fluid (VMG II) [38], serially diluted, and all plated onto mitis salivarius-bacitracin agar (mitis salivarius agar (Acumedia Man Inc., Baltimore, MD, USA), Chapman Tellurite solution (Difco Lab Inc., Detroit, MI, USA), 150 g sucrose and 200 U/ml bacitracin (Sigma Diagnostics, St. Louis, MO, USA) [39]). After incubation at 37°C for 48 h in candle jars, the colonies were counted. It is low if <10^5^ CFU/ml, moderate if between 10^5^ and 10^6^ CFU/ml, and high if ≥10^6^ CFU/ml.
- *Lactobacillus count*. Saliva (1 ml) was placed in 3 ml of transport fluid (VMG II) [38], serially diluted, and all plated onto Rogosa agar (Merck KGaA, Darmstadt, Germany) [40]. After incubation at 37°C for 48 h in candle jars, the colonies were counted. It is low if <10^4^ CFU/ml, moderate if between 10^4^ and 10^5^ CFU/ml, and high if ≥10^5^ CFU/ml.

Descriptive statistics, including means, standard deviations, and minimum and maximum values, were calculated for all variables using the Statistical Package for Social Sciences (IBM Corporation, Armonk, NY, USA). Wilcoxon rank and Mann-Whitney *U* tests were used to determine intragroup and intergroup differences as regards qualitative data. To compare quantitative data between the groups, chi-square and Fisher's exact tests were undertaken, while intragroup differences were tested with McNemar test. The level of statistical significance was set at *p* < 0.05.

## Results and discussion

Table 1 shows that, at the baseline registration of the data, no statistically significant differences were found between the two groups in the DMFT index, PI, GBI values, salivary flow rate, buffering capacity, and *S*. *mutans* and *Lactobacillus* counts (Mann-Whitney *U* and Fisher's exact tests). This means that the two samples were homogeneous and comparable.

**Table 1:** **Baseline registration of data**

		Lingual technique	Labial technique	***P*** value
		(***n*** = 10)	(***n*** = 10)	
Oral hygiene	DMFT index (mean ± SD)	2.00 ± 2.16	1.90 ± 2.18	NS
	Plaque index (mean ± SD)	0.47 ± 0.18	0.42 ± 0.17	NS
	Gingival bleeding index (mean ± SD)	0.18 ± 0.13	0.31 ± 0.21	NS
Salivary flow rate	ml/min (mean ± SD)	1.11 ± 0.47	1.06 ± 0.65	NS
Buffering capacity	pH (mean ± SD)	5.20 ± 0.79	5.35 ± 0.41	NS
*S*. *mutans*	Low *n* (%)	0 (0)	0 (0)	NS
	Moderate *n* (%)	7 (70)	5 (50)	
	High *n* (%)	3 (30)	5 (50)	
*Lactobacillus*	Low *n* (%)	4 (40)	2 (20)	NS
	Moderate *n* (%)	3 (30)	5 (50)	
	High *n* (%)	3 (30)	3 (30)	

Oral hygiene, salivary markers, and distribution of patients according to various levels of *S*. *mutans* and *Lactobacillus*. NS, not significant (Mann-Whitney *U* and Fisher's exact tests).

Wilcoxon rank test revealed a statistically significant increase between the data of *T*0 and *T*1 and the data of *T*0 and *T*2 of the PI scores (*p* < 0.05) and between *T*0 and *T*2 of the GBI scores (*p* < 0.05) in the group treated with lingual appliance. In the group treated with labial appliance, the GBI values increased significantly between *T*0 and *T*1 (*p* < 0.05) and decreased significantly between *T*1 and *T*2 (*p* < 0.01). No statistically significant differences were found at all time points in the two treatment groups as regards the salivary flow rate and saliva buffer capacity (pH). These results are reported in Table 2.

**Table 2:** **Intragroup differences in plaque index**, **gingival bleeding index scores**, **and salivary markers**

		Plaque index (mean ± SD)	Gingival bleeding index (mean ± SD)	Saliva flow rate (ml/min, mean ± SD)	Buffering capacity (pH, mean ± SD)
Lingual technique (*n* = 10)	*T*0	0.47 ± 0.18	0.18 ± 0.13	1.11 ± 0.47	5.20 ± 0.79
	*T*1	0.56 ± 0.15	0.22 ± 0.07	1.25 ± 0.38	5.30 ± 0.85
	*T*2	0.59 ± 0.16	0.29 ± 0.19	1.39 ± 0.46	5.00 ± 0.88
	*T*0-*T*1	*	NS	NS	NS
	*T*1-*T*2	NS	NS	NS	NS
	*T*0-*T*2	*	*	NS	NS
Labial technique (*n* = 10)	*T*0	0.42 ± 0.17	0.31 ± 0.21	1.06 ± 0.65	5.35 ± 0.41
	*T*1	0.52 ± 0.25	0.45 ± 0.17	1.32 ± 0.45	5.75 ± 0.79
	*T*2	0.43 ± 0.20	0.33 ± 0.13	1.50 ± 1.22	5.50 ± 0.41
	*T*0-*T*1	NS	*	NS	NS
	*T*1-*T*2	NS	**	NS	NS
	*T*0-*T*2	NS	NS	NS	NS

NS, not significant. *p < 0.05, **p < 0.01 (Wilcoxon rank test).

The McNemar test showed a statistically significant increase in *S*. *mutans* counts between *T*0 and *T*2 (*p* < 0.05) in the saliva samples of patients treated with lingual appliance, while it found no differences in the labial group. No statistically significant differences were found at the three time points in the two treatment groups as regards the *Lactobacillus* counts (Table 3).

**Table 3:** **Intragroup differences in the distribution of patients according to various levels of** ***S***. ***mutans*** **and** ***Lactobacillus***

		***S***. ***mutans***			***Lactobacillus***		
		Low ***n*** (%)	Moderate ***n*** (%)	High ***n*** (%)	Low ***n*** (%)	Moderate ***n*** (%)	High ***n*** (%)
Lingual technique (*n* = 10)	*T*0	0 (0)	7 (70)	3 (30)	4 (40)	3 (30)	3 (30)
	*T*1	0 (0)	2 (20)	8 (80)	2 (20)	2 (20)	6 (60)
	*T*2	0 (0)	1 (10)	9 (90)	2 (20)	2 (20)	6 (60)
	*T*0-*T*1	NS	NS	NS	NS	NS	NS
	*T*1-*T*2	NS	NS	NS	NS	NS	NS
	*T*0-*T*2	*	*	*	NS	NS	NS
Labial technique (*n* = 10)	*T*0	0 (0)	5 (50)	5 (50)	2 (20)	5 (50)	3 (30)
	*T*1	0 (0)	2 (20)	8 (80)	3 (30)	4 (40)	3 (30)
	*T*2	0 (0)	2 (20)	8 (80)	1 (10)	5 (50)	4 (40)
	*T*0-*T*1	NS	NS	NS	NS	NS	NS
	*T*1-*T*2	NS	NS	NS	NS	NS	NS
	*T*0-*T*2	NS	NS	NS	NS	NS	NS

NS, not significant. *p < 0.05 (McNemar test).

The comparison between the two groups with Mann-Whitney *U* test demonstrated no statistically significant differences at any time point as regards PI, salivary flow rate, and saliva buffer capacity (pH). Only the GBI value at *T*1 term was significantly higher (*p* < 0.01) in the patients treated with labial appliance (Table 4).

**Table 4:** **Intergroup differences of PI and GBI scores**, **salivary markers**

	Plaque index			Gingival bleeding index			Salivary flow rate (ml/min)			Buffering capacity (pH)		
	***T***0 (mean ± SD)	***T***1 (mean ± SD)	***T***2 (mean ± SD)	***T***0 (mean ± SD)	***T***1 (mean ± SD)	***T***2 (mean ± SD)	***T***0 (mean ± SD)	***T***1 (mean ± SD)	***T***2 (mean ± SD)	***T***0 (mean ± SD)	***T***1 (mean ± SD)	***T***2 (mean ± SD)
Lingual technique (*n* = 10)	0.47 ± 0.18	0.56 ± 0.15	0.59 ± 0.16	0.18 ± 0.13	0.22 ± 0.07	0.29 ± 0.19	1.11 ± 0.47	1.25 ± 0.38	1.39 ± 0.46	5.20 ± 0.79	5.30 ± 0.85	5.00 ± 0.88
Labial technique (*n* = 10)	0.42 ± 0.17	0.52 ± 0.25	0.43 ± 0.20	0.31 ± 0.21	0.45 ± 0.17	0.33 ± 0.13	1.06 ± 0.65	1.32 ± 0.45	1.50 ± 1.22	5.35 ± 0.41	5.75 ± 0.79	5.50 ± 0.41
*p* value	NS	NS	NS	NS	**	NS	NS	NS	NS	NS	NS	NS

NS, not significant. **p < 0.01 (Mann-Whitney *U* test).

The comparison between the two groups with Fisher's exact test demonstrated no statistically significant differences at any time point in the *Lactobacillus* and *S*. *mutans* counts (Tables 5 and 6).

**Table 5:** **Intergroup differences in the distribution of the patients according various levels of** ***Lactobacillus***

	***Lactobacillus***								
	***T***0			***T***1			***T***2		
	Low ***n*** (%)	Moderate ***n*** (%)	High ***n*** (%)	Low ***n*** (%)	Moderate ***n*** (%)	High ***n*** (%)	Low ***n*** (%)	Moderate ***n*** (%)	High ***n*** (%)
Lingual technique (*n* = 10)	4 (40)	3 (30)	3 (30)	2 (20)	2 (20)	6 (60)	2 (20)	2 (20)	6 (60)
Labial technique (*n* = 10)	2 (20)	5 (50)	3 (30)	3 (30)	4 (40)	3 (30)	1 (10)	5 (50)	4 (40)
*p* value	NS	NS	NS	NS	NS	NS	NS	NS	NS

NS, not significant (Fisher's exact test).

**Table 6:** **Intergroup differences in the distribution of the patients according various levels of** ***S***. ***mutans***

	***S***. ***mutans***								
	***T***0			***T***1			***T***2		
	Low ***n*** (%)	Moderate ***n*** (%)	High ***n*** (%)	Low ***n*** (%)	Moderate ***n*** (%)	High ***n*** (%)	Low ***n*** (%)	Moderate ***n*** (%)	High ***n*** (%)
Lingual technique (*n* = 10)	0 (0)	7 (70)	3 (30)	0 (0)	2 (20)	8 (80)	0 (0)	1 (10)	9 (90)
Labial Technique (*n* = 10)	0 (0)	5 (50)	5 (50)	0 (0)	2 (20)	8 (80)	0 (0)	2 (20)	8 (80)
*p* value	NS	NS	NS	NS	NS	NS	NS	NS	NS

NS, not significant (Fisher's exact test).

## Discussion

Young adults, of typical age and gender distribution of lingual orthodontic patients [41, 42], were chosen as good candidates to wear lingual appliances (1) because they are more interested in esthetic appliances and then (2) because in children and adolescents, some teeth are partially erupted and not good for taking precise impressions for lingual setup and indirect bonding. Moreover, in the literature, we can find many articles analyzing various similar ranges of age [17, 22, 34, 43, 44]. The majority of patients enrolled in the present study were females less than 23 years of age. Stadelmann et al. found that almost a quarter of the population undergoes an orthodontic treatment, half of it with a mean age of 15 to 24 years old [45].

As regards intragroup differences, the significant increases in PI and GBI values from *T*0 to *T*2 in the lingual orthodontic group could be explained by the fact that plaque deposits on the lingual gingival margin of the teeth are more difficult to remove with standard oral hygiene procedures with respect to the labial side; if maintained for a long time, they represent a bacterial injury for the gingiva and can cause gingival inflammation [46].

Some studies in the literature confirm our results: Stamm et al. [34] found that, during lingual orthodontic treatment, there is oral hygiene impairment, even if data were collected afterwards by a questionnaire and the authors did not specify which parameter of oral hygiene they considered. Also, Artun [32] reported that visible plaque and gingivitis were present in up to 70% of the patients after starting the lingual treatment. Sinclair et al. [31] saw significantly elevated plaque accumulation in patients wearing lingual brackets.

According to many retrospective [29, 31, 32] and prospective studies [33, 34] of the literature, wider lingual brackets cause a reduced interbracket distance and make oral hygiene procedures very difficult [1, 26]–[28], with consequent risk for plaque accumulation and g**i**ngivitis. Many researchers in their studies declared that brackets and their components create a favorable condition for rapid plaque accumulation, decreasing of plaque pH, enamel demineralization, and incipient carious lesions [6, 47]–[49]. Øgaard et al. [10] and Yun-Wah Lau et al. [5] found that fixed orthodontic appliances make oral hygiene difficult and cause plaque retention even for the most motivated patients, and almost all of them experience some degree of gingival inflammation. Gingival recession and loss of alveolar bone, although multifactorial in origin [50], have been reported as one of the common sequelae of orthodontic procedures, especially when the teeth move in the presence of inflammation [5, 19].

Now, comparing the two groups of patients in our study, we can see that the lingual group demonstrates more difficulty in removing food and plaque deposits around the brackets, as confirmed by the literature [33, 34], even if we found that the labial group patients experienced a transient gingivitis at *T*1 that was completely resolved at *T*2. Therefore, oral hygiene instruction would be essential in all cases of orthodontic treatment, and the use of adjuncts such as sonic electric toothbrushes, interproximal brushes, chlorhexidine mouthwashes, fluoride mouthwashes, and regular professional cleaning should be reinforced [5]. However, patient motivation and dexterity are paramount in the success.

Saliva is an important modifying factor in the cariogenic potential of dental plaque; thus, it should be considered in the orthodontic treatment planning because the composition, pH, and flow rate may influence bacterial adherence to enamel and orthodontic surfaces. According to Jensen and Bratthall [12], the number of *S*. *mutans* in saliva can be used for the evaluation of individual caries risk. An augmentation in stimulated salivary flow, buffer capacity, and salivary pH after placement of orthodontic appliances would be considered as protective factor.

In contrast, a rise in the levels of *S*. *mutans* and *Lactobacillus* counts is a negative risk factor to the oral environment [18]. The most common method used to identify caries-susceptible people is estimating the number of cariogenic bacteria such as *Lactobacillus* and *S*. *mutans* in saliva or plaque samples [12, 18, 20, 22].

As regards salivary parameters, in this study, salivary flow rate and buffer capacity did not differ significantly from the baseline to the two time points. However, we observed a significant increase in the number of patients with high levels of *S*. *mutans* colonization between *T*0 and *T*2 in the group with lingual device.

In a study with patients undergoing labial appliance treatment, Rosenbloom and Tinanoff [23] observed an increase of microbial oral population, particularly of *S*. *mutans*, known as the etiologic agent of dental caries [16, 51]. On the other hand, Lara-Carrillo et al. [18] found that the placement of orthodontic appliances promoted a major stimulated salivary flow and buffer capacity in the subjects with significant differences in the salivary production before treatment and after debonding of the appliance. Nevertheless, patients showed a slight increase in the levels of *S*. *mutans* and *Lactobacillus*. Interestingly, Gorelick et al. [21] found no incidence of white spot formation associated with lingual-bonded retainers, which would suggest that salivary buffering capacity and flow rate have a role in protection against acid attack.

## Conclusions

Lingual and labial orthodontic appliances showed a different potential in modifying the investigated clinical parameters; in accordance with many studies in the literature, we found out that patients wearing STb lingual orthodontic appliance had more plaque retention 4 and 8 weeks after bonding, more gingival inflammation, and more *S*. *mutans* counts 8 weeks after bonding. On the contrary, no differences were found between the two groups as regards the *Lactobacillus* counts, the salivary flow rate, and the saliva buffer capacity. Further research with larger patient samples and longer follow-up time are needed.
